# Prevalence of impaired renal function and determinants in the southwest of Iran

**DOI:** 10.1186/s12882-021-02484-x

**Published:** 2021-08-10

**Authors:** Saba Alvand, Farhad Abolnezhadian, Sudabeh Alatab, Zahra Mohammadi, Fatemeh Hayati, Mohammad Noori, Leila Danehchin, Yousef Paridar, Bahman Cheraghian, Zahra Rahimi, Sanam Hariri, Sahar Masoudi, Seyed Ali Mard, Ali Akbar Shayesteh, Hossein Poustchi

**Affiliations:** 1 Liver and Pancreatobiliary Diseases Research Center, Digestive Diseases Research Institute, Tehran University of Medical Sciences, Tehran, IR Iran; 2 Shoshtar Facullty of Medical Sciences, Shoshtar, Iran; 3 Ahvaz Jundishapur University of Medical Sciences, Ahvaz, IR Iran; 4 Digestive Disease Research Center, Digestive Disease Research Institute, Tehran University of Medical Sciences, Tehran, IR Iran; 5 Chronic Renal Failure Research Center, Imam khomeini Hospital, Ahvaz Jundishapur Uiversity of Medical Science, Ahvaz, IR Iran; 6 Abadan Faculty of Medical Sciences, Abadan, IR Iran; 7 Behbahan Faculty of Medical Sciences, Behbahan, IR Iran; 8 School of Medicine, Dezful University of Medical Sciences, Dezful, IR Iran; 9 Department of Biostatistics and Epidemiology, Alimentary Tract Research Center, Imam Khomeini Hospital Clinical Research Development Unit, School of Public Health, Ahvaz Jundishapur University of Med, Ahvaz, IR Iran; 10 Hearing Research Center, Department of Biostatistics and Epidemiology, School Of Public Health, Ahvaz Jundishapur University Of Medical Sciences, Ahvaz, IR Iran; 11 Alimentary Tract Research Center, Imam Khomeini Hospital Clinical Research Development Unit, the School of Medicine, Ahvaz Jundishapur University of Medical Sciences, Ahvaz, IR Iran; 12 Alimentary Tract Research Center, Imam Khomeini Hospital, Clinical Research Development Unit, Faculty of Medicine, Ahvaz Jundishapur University of Medical Sciences, Ahvaz, IR Iran

**Keywords:** Chronic kidney disease, Determinants, Prevalence, Iran

## Abstract

**Background:**

Chronic kidney disease (CKD) is a growing global health problem with faster progression in developing countries such as Iran. Here we aimed to evaluate the prevalence and determinants of CKD stage III+.

**Methods:**

This research is part of the Khuzestan Comprehensive Health Study (KCHS), a large observational population-based cross-sectional study in which 30,041 participants aged 20 to 65 were enrolled. CKD was determined with estimated glomerular filtration rate (eGFR) less than 60 ml/min/1.73m^2^, based on two equations of Modification of Diet in Renal Disease (MDRD) and Chronic Kidney Disease Epidemiology Collaboration (CKD-EPI). The multivariate logistic regression was used to evaluate the CKD stage III+ determinants.

**Results:**

Prevalence of CKD stage III+ is estimated to be 7.1, 5.5, and 5.4% based on MDRD, CKD-EPI, and combination of both equations, respectively. More than 89% of CKD subjects aged higher than 40 years. In regression analysis, age more than 40 years had the strongest association with CKD stage III+ probability (OR: 8.23, 95% CI: 6.91–9.18). Higher wealth score, hypertension, High-Density Lipoprotein levels less than 40 mg/dl, and higher waist to hip ratio were all associated with CKD stage III+ while Arab ethnicity showed a protective effect (OR: 0.69, 95% CI: 0.57–0.78).

**Conclusion:**

Our findings provide detailed information on the CKD stage III+ and its determinants in the southwest region of Iran. Due to strong association between age and CKD stage III+, within a few decades we might expect a huge rise in the CKD prevalence.

**Supplementary Information:**

The online version contains supplementary material available at 10.1186/s12882-021-02484-x.

## Background

Chronic kidney disease (CKD) is a general term for a wide spectrum of kidney diseases identified with progressive loss of functional nephrons [1]. It is a major health problem with a growing epidemic dimension worldwide. Based on the most recent analysis of the Global Burden of Disease study 2017, almost 700 million people suffer from CKD, globally [2]. Besides, to be an important contributor to morbidity and mortality in societies, CKD may finally progress toward end-stage renal disease (ESRD), a condition in which patients cannot survive unless renal replacement modalities (dialysis or transplant) being provided for them. Renal replacement therapies could place a huge financial burden on the health system especially in countries with limited financial resources. For example, the total cost of ESRD program in the United State was approximately $35.9 billion in 2017 [3]. In addition to the financial burden, life expectancy is substantially compromised in subjects suffering from ESRD [4]. Early diagnosis and appropriate treatment of CKD, when the disease is at mild to moderate stages, could slow down or even stop the progression toward ESRD [5–8]. Published data indicates that increase in CKD prevalence is affecting more the developing countries; the annual change in CKD prevalence in developed countries shows a decreasing trend, while the figure is increasing in developing nations [2, 9].

In our country, large population-based studies are spares and the surveys have shown marked heterogeneity of CKD prevalence in the general population. In a study by Najafi et al., the prevalence of CKD stage III+ was reported to be 8.9% [10]. More recently, based on the Golestan Cohort Study, Sepanlou et al. reported the CKD stage III+ prevalence at 23.8% in ages higher than 40 years [11]. Whether this increase is highlighting a real increase in CKD prevalence or other factors including the study type contributed to these findings, need to be clarified.

GFR estimation (eGFR) using endogenous filtration markers such as serum creatinine has been used routinely both in clinic and population studies as a measure of global kidney function. The Modification of Diet in Renal Disease (MDRD) and CKD Epidemiology Collaboration (CKD-EPI) are the two most employed equations for GFR estimation. These two methods are developed based on rigorous methods and have been evaluated in large and diverse population. They are regarded as the most practical GFR estimation equations available for general studies [8, 12, 13]. Some studies have suggested that the CKD-EPI equation might be more accurate than the MDRD study equation particularly in populations without CKD [8, 14], although debates are still open [15, 16].

The large population-based studies evaluating the prevalence of CKD and its risk factors are short in our country and most of health policy decision-making is based on data from other nations with sufficient data. The need for performing high-quality population-based studies on CKD is an inevitable fact. In our country, a large cross-sectional study called “Khuzestan Comprehensive Health study (KCHS)” has been performed in the southwest region of Iran, in which more than 30,000 adult individuals were recruited [17]. The main objective of the KCHS study was to collect substantial information on the health status of people living in this region who have different environmental and socioeconomic situations compared to other parts of Iran. Here in this study, we investigated the prevalence of CKD stage III+ and analyzed the factors affecting the eGFR by employing data obtained from this survey. To be more precise we assessed the renal function estimation obtained by both CKD-EPI and MDRD equations and analyzed the data accordingly.

## Methods

### Population and study design

Located in southwest of Iran, Khuzestan province is the major oil-producing region in our country. The natural condition, geological climate and soil characteristics of this region is unique. The extreme high temperature (possibly one of the hottest places on earth), frequent dust storms, air pollution as well as soil contamination caused by oil production heavily affect the health status of its residents. The capital city, Ahvaz, hits one of the highest temperature records in the world; in summer, the temperature can reach to 50 °C. Moreover, the region was highly damaged during the Iran-Iraq war (1980–1988), which further hampered the physical and psychological health of its residents.

Khuzestan is also known for its ethnic diversity. The population of Khuzestan consists of different ethnicity: Fars, Arabs, Bakhtiarys, and Lurs are the main ethnic groups. The socioeconomic status and life- style are quite different among these ethnicities. Arabs are mostly in lower socioeconomical levels of the society. Their diet comprises of different spices and carbohydrates rich- foods. On the other hand, Fars mostly resides in urban areas and rice is usually their main dish. Interestingly, according to central bank of Islamic republic of Iran, Khuzestan has obtained the highest budget in Iran among all the provinces (https://irandataportal.syr.edu/annual-budgets). The history of war, extremely hot weather, time to time air pollution, multi-ethnic culture, and unavailability of previous health status despite enormous budget, lead us to select this province for comprehensive health survey.

The study was conducted from 2016 to 2019 as part of KCHS study. The details of the KCHS have been described elsewhere [17]. National Institute for Medical Research Development (NIMAD) and the Iranian Blood Transfusion Organization (IBTO) funded this study and the Digestive Diseases Research Institute (DDRI) in association with Jundishapur, Abadan, Dezful, and Behbahan medical universities executed it. The protocol was approved by the ethics committee of NIMAD (IR.NIMAD.REC.1394.002), and written informed consent was obtained from all participants. Using a multistage random sampling, the 1079 random Health Houses across 27 counties in the province were selected, and then 30 eligible individuals were randomly selected from the population covered by that Health House. The inclusion criteria set as both sexes, aged between 20 to 65 years. The exclusion criteria were individuals with mental, psychological, or physical disabilities that could not respond or attend the interview, unwillingness to participate, and being a temporary resident in the province.

The data were collected by employing a questionnaire through interviews. The collected data included demographic, socioeconomic, physical activity (International Physical Activity Questionnaires (IPAQ) [18], existing major diseases, medication history, and lifestyle risk factors (Appendix). A one-time blood sample was collected from all participants after 8–12 h of fasting. The blood samples were transferred to the reference laboratory within 3 h of sampling for measuring the fasting blood sugar (FBS), creatinine (Cr), total cholesterol (TC), triglyceride (TG), and high-density lipoprotein (HDL). The sample was analyzed by BT 1500 autoanalyzer (Biotecnica Instruments, Italy) using commercial kits (Pars Azmun, Iran). Due to the large number of participants, collecting first morning urine sample and transferring to reference laboratory within 2 h were not possible, therefore the urine samples were not analyzed in this study.

Trained health personnel with similar standard tools in each center measured height, waist circumferences, and weight by the Seca 206 body meter measuring tape and adjusted Seca 762 mechanical flat scale in kilograms, respectively. Blood pressure was measured after 5 min of rest and in a sitting position, twice from each arm with 10 min interval using the Riester auscultatory Sphygmomanometers [19]. The calculated average systolic and diastolic blood pressure were taken as mean systolic and diastolic blood pressures, respectively.

### Definitions

Hypertension (HTN) was defined as having any of the following conditions: self-reporting of HTN, anti-hypertensive medication consumption, systolic blood pressure (SBP) ≥ 140 mmHg, diastolic blood pressure (DBP) ≥ 90 mmHg [19].

Diabetes Mellitus was (DM) defined as having any of the following conditions: self-reporting of DM, blood glucose-lowering medications consumption, FBS ≥ 126 mg/dl [20].

Hypercholesterolemia was defined as having any of the following conditions: cholesterol-lowering medications consumption, TC > = 200 mg/dl [21].

Metabolic syndrome was based on ATP III criteria and Iranian criteria (at least three items of the following conditions) [22–24]:

- FBS ≥100 mg/dl or having Diabetes Mellitus
- SBP > =130 mmHg or DBP > =85 mmHg
- TG > 150 mg/dl or consuming triglyceride -lowering medications
- HDL < 40 mg/dL in men or < 50 mg/dL in women or consuming medications
- Waist circumference ≥ 95 cm in both sexes

Physical activity status included in the analysis as low, middle, and high activity by metabolic equivalent task (MET) score [18].

Socioeconomic status included in the analysis as very low, low, middle, and high based on a validated questionnaire [17].

### Variable related to kidney function

 Serum creatinine levels were measured according to the standard colorimetric Jaffe-Kinetic reaction method. The assay was not traceable to isotope dilution mass spectroscopy (IDMS). The urine analysis was not assessed in this study.

MDRD study equation and CKD-EPI equation were used to estimate GFR [8, 14], based on the following formula:

1. GFR by MDRD (ml/min/1.73 m^2^) = 176 × Cr^-1.154^ × age^-0.203^ × 0.742 (if female)
2. GFR by CKD-EPI (ml/min/1.73 m^2^) = A × (Cr / B) ^C^ × 0.993^age^: A, B, C substituted as following:

**Table:** 

	**Female**		**Male**	
**Cr (mg/dl)**	**≤0.7**	**> 0.7**	**≤0.9**	**> 0.9**
A	144	144	141	141
B	0.7	0.7	0.9	0.9
C	−0.329	−1.209	− 0.411	− 1.209

CKD stage III+ was based on one measurement of serum creatinine and defined as: eGFR less than 60 ml/min/1.73 m^2^ by applying both equations.

eGFR was divided into following stages [25]: CKD stages III: eGFR between 30 and 59 ml/min per 1.73 m^2^, CKD stage IV: eGFR between 30 and 15 ml/min per 1.73 m^2^, CKD stage V: eGFR less than 15 ml/min per 1.73 m^2^. *Normal eGFR was* defined as having eGFR higher than 90 ml/min/1.73 m^2^ (due to unclear kidney damage in this spectrum without urine data)*.*

### Statistical analysis

We specified the frequency, mean, and standard deviation of the variables then investigated for the relation of qualitative variables. Physical activity was categorized into low, middle, and high activity by MET score [18]. ,Wealth score was grouped into quartiles called very low, low, middle, and high [17]. We used the cross tabulation to investigate the association between CKD stage III+ and categorized variables and student t-test to assess the association between CKD stage III+ and quantitative variables. A logistic regression model with impaired renal function as the outcome of interest was used to investigate the odds ratio of each variable. All variables with *p*-value less than 0.1 in former cross-tabulations were included in multivariable analysis. All the analyses were carried out with SPSS version 25 and the statistical significance was declared if the p-value was less than 0.05.

## Results

During the study period, 30,506 participants were enrolled, from which 30,041 subjects had blood samples and therefore included in the study. The participants who refused to participate in blood sampling were mostly rural resident (2.1% vs. 1.1% in urban areas, p < 0.001), and Bakhtiarys (1.6% in Bakhtiarys, 1.4% in Arabs, and 0.9% in Fars, p = 0.006). There was no significant difference in age, sex, and medical issues of non-participants compared to others. The crude prevalence of MDRD based and CKD- EPI based CKD stage III+ in our population were 7.1% (n = 2135) and 5.5% (n = 1651), respectively. Based on our definition of CKD, about 5.4% of subjects (n = 1640) had CKD stage III+ and 12,963 participants defined as subjects with normal eGFR. Table 1 shows the detailed characteristics of these groups. The mean age of subjects in the CKD stage III+ group was 53.1 ± 9.2 years which was significantly higher than both total and normal eGFR groups. Prevalence of CKD stage III+ by age categorization showed an elevation pattern *in which by increasing the age the prevalence of CKD was increased. This pattern was observed in both sexes*. About 15.4% of total subjects had DM, while this value was about twice higher in the CKD stage III+ group. The prevalence of hypertension in the CKD stage III+ group (43.6%) was significantly higher than both total (20.1%) and normal eGFR groups (14.7%).

**Table 1:** The characteristics of the total, CKD stage III+, normal eGFR groups in our study

Parameters		Total group (***N*** = 30,041)	CKD stage III + - group (N = 1640)	Normal eGFR (***N*** = 12,963)	***P*** value
Age (year)	mean (SD)	41.7(11.9)	53.1(9.2)	38.3(11.7)	< 0.01
	< 40, n (%)	13,844(46.1)	172(10.5)	7659(59.1)	
	≥ 40, n (%)	16,196(53.9)	1467(89.5)	5304(40.9)	
Gender, n (%)	Male	10,748(35.8)	658(40.1)	4769(36.8)	< 0.01
	Female	19,293(64.2)	982(59.9)	8194(63.2)	
Marital status, n (%)	Married	24,930(83)	1417(86.4)	10,453(80.6)	< 0.01
	Single	5111(17)	223(13.6)	2510(19.4)	
Education, n (%)	Illiterate	5505(18.3)	455(27.7)	2185(16.9)	< 0.01
	School	16,454(54.8)	871(53.1)	7242(55.9)	
	Diploma, higher	8079(26.9)	314(19.1)	3535(27.3)	
Ethnicity, n (%)	Fars	5558(18.5)	364(22.3)	1931(14.9)	< 0.01
	Arab	14,699(49)	755(46.2)	74.3(57.2)	
	Bakhtiari	6613(22.1)	370(22.7)	2494(19.3)	
	others	3115(10.4)	144(8.8)	1119(8.6)	
Smoking, n (%)	No	26,734(89.2)	1414(86.4)	11,578(89.4)	< 0.01
	Yes	3251(10.8)	222(13.6)	1370(10.6)	
Wealth score, n (%)	Very low	7513(25.2)	324(20)	3584(27.8)	< 0.01
	Low	7390(24.7)	403(24.8)	3111(24.1)	
	Middle	7983(26.7)	478(29.4)	3434(26.6)	
	High	6978(23.4)	419(25.8)	2764(21.4)	
Physical activity n (%)	Low	9302(30)	580(35.5)	3974(30.7)	< 0.01
	Medium	12,853(42.9)	698(42.7)	5491(42.5)	
	High	7810(26.1)	355(21.7)	3462(26.8)	
DM, n (%)	No	25,372(84.6)	1188(72.6)	11,236(89.8)	< 0.01
	Yes	4616(15.4)	449(27.4)	1713(13.2)	
HTN, n (%)	No	23,910(79.9)	921(56.4)	11,029(85.3)	< 0.01
	Yes	6015(20.1)	712(43.6)	1901(14.7)	
IHD, n (%)	No	29,584(98.5)	1582(96.5)	12,828(99)	< 0.01
	Yes	457(1.5)	58(3.5)	135(1)	
Hyperchol n (%)	No	17,589(58.5)	816(49.8)	8137(62.8)	< 0.01
	Yes	12,452(41.5)	824(50.2)	4826(37.2)	
MS, n (%)	No	20,323(67.7)	840(51.2)	9447(72.9)	< 0.01
	Yes	9718(32.3)	800(48.8)	3516(27.1)	
HDL n (%)	< 40 mg/dl	4873(16.2)	333(20.3)	2067(15.9)	< 0.01
	≥ 40 mg/dl	25,168(83.8)	1307(79.7)	10,896(84.1)	
BMI (kg/m^2^), mean (SD)		27.6 (5.3)	28.6(5.1)	27.1(5.5)	< 0.01
WC (cm), mean (SD)		9217(14.6)	96.9(14.1)	90.4(14.8)	< 0.01
WHtR, mean (SD)		0.57(0.09)	0.6(0.09)	0.55(0.09)	< 0.01
WHR mean (SD)		0.89(0.08)	0.93(0.08)	0.88(0.08)	< 0.01

The prevalence of different stages of CKD based on each equation is provided in Table 2. Most CKD subjects were categorized in CKD stage IIIa regardless of the type of applied eq. (89.3% by MDRD, and 89.3% by CKD-EPI) in both genders. The prevalence of CKD was slightly higher in women compared to men in MDRD-based CKD (6.82% in men versus 7.27% in women).

**Table 2:** Prevalence of CKD stages based on MDRD and CKD-EPI equations

CKD stage n (%)	MDRD			CKD- EPI		
	All	Male	Female	All	Male	Female
Stage III	2074 (6.91)	704 (6.55)	1370 (7.1)	1602 (5.34)	650 (6.05)	952 (4.94)
Stage IV	42(0.14)	16(0.15)	25(0.13)	35(0.12)	14(0.13)	22(0.11)
Stage V	19(0.06)	13(0.12)	7(0.04)	14(0.05)	10(0.09)	3(0.02)

CKD was defined as eGFR < 60 ml/min/1.73 m2

### Determinants of CKD

Results of the univariate and multivariate logistic regression models are shown in Table 3.

**Table 3:** Risk Factors in Univariate and Multivariable Logistic Regression Model for Chronic Kidney Disease stage III+

Characteristics		Univariate analysis	Multivariate analysis
Gender	Female	Reference	Reference
	Male	1.15(1.03–1.27)	0.88 (0.78–1.01)
Age	< 40	Reference	Reference
	≥40	12.32 (10.48–14.48)	**8.23 (6.91–9.81)**
Residency	Rural	Reference	–
	Urban	1.36(1.21–1.54)	–
Wealth score	Very low	Reference	Reference
	Low	1.43(1.22–1.67)	**1.48 (1.25–1.75)**
	Middle	1.54(1.32–1.78)	**1.65 (1.41–1.94)**
	High	1.67(1.43–1.95)	**1.50 (1.27–1.78)**
Ethnicity	Fars	Reference	Reference
	Arab	0.54(0.47–0.61)	**0.67 (0.57–0.78)**
	Bakhtiary	0.78(0.67–0.92)	0.91 (0.77–1.08)
	Others	0.68(0.55–0.83)	**0.77 (0.62–0.97)**
BMI	Normal	Reference	Reference
	Underweight	0.34(0.19–0.58)	0.59 (0.33–1.05)
	Overweight	1.67(1.46–1.91)	0.99 (0.85–1.15)
	Obesity	1.99(1.73–2.28)	0.85 (0.73–1.01)
Physical activity	Low	Reference	–
	Medium	0.87(0.77–0.98)	–
	High	0.70(0.61–0.80)	–
HDL level (mg/dl)	> = 40	Reference	Reference
	< 40	1.34(1.18–1.52)	**1.27 (1.09–1.47)**
Hypertension		4.48(4.02–5.00)	**2.18 (1.93–2.46)**
Ischemic heart disease		3.48 (2.55–4.76)	–
Diabetes Mellitus		2.47(2.19–2.79)	–
Hypercholesterolemia		1.70(1.53–1.88)	1.11 (0.98–1.24)
Metabolic syndrome		2.55(2.30–2.84)	–
Smoking		1.32(1.14–1.54)	–
WC		1.59 (1.51–1.68)	–
WHtR		1.64 (1.56–1.73)	–
WHR		1.84 (1.74–1.94)	1.31 (1.22–1.40)

All variables with p-value less than 0.1 in cross-tabulation, included in multivariable analysis

*HDL* High-density Lipoprotein, *BMI* Body Mass Index, *WC* Waist circumference, *WHtR* Waist to height ratio, *WHR* Waist to hip ratio

All the included variables had a significant association with CKD stage III+ in the crude model. Applying the adjusted model showed that older age (higher than 40 years versus lower than 40 years) had the greatest association with CKD stage III+, followed by HDL levels less than 40 mg/dl. Other variables that had significant association with CKD stage III+ in our model included: wealth score (low, middle, and high versus very low), hypertension, and waist to hip ratio. BMI categories, and history of ischemic heart disease lost their association with CKD stage III+ in multivariate analysis. However, the Arab ethnicity (versus Fars) exhibited a significant reverse association with CKD stage III+ both in crude and multivariate model.

## Discussion

CKD is a major public health problem that its prevalence is increasing. In the present cross-sectional large population-based study which was carried out for the first time in southwest of Iran, we assessed the prevalence of CKD stage III+ (eGFR< 60 ml/min/1.73 m^2^) among more than 30,000 participants and revealed that 5.5% of participants had CKD stage III+ when their kidney function was assessed by both MDRD and CKD- EPI equations. While 2013 KDIGO recommends that action plan and clarification of prognosis in CKD subjects be based on categorization of eGFR in G1 to G5 and albuminuria in A1 to A3, here is in this study, we used the KIDGO 2005 CKD classification [25], because of not having the urine data and also because of epidemiological essence of the study. We cannot be sure about the renal function of participants with eGFR more than 90 ml/min/1.73 m^2^, but as long as the comparison was based on the eGFR, we grouped participants into two absolute groups of “normal eGFRs” and “CKD stage III+”. Although several studies evaluated the prevalence of CKD in different parts of our country, there are great disparities regarding reported CKD prevalence among them. In Safarinejad and colleagues’ study, in a total of 17,240 persons of either sex over 14 years old from 30 counties, the CKD stage III+ prevalence was found to be 8.3% [26]. Mahdavi-mazdeh and colleagues evaluated the kidney function in more than 30,000 Taxi drivers, as part of a “large-scale cross-sectional survey of Kidney Disease Screening of Taxi Drivers in Tehran” and found a CKD prevalence close to 6.5% [27]. On the other hand, Najafi et al. and Barahimi et al. studies reported lower prevalence of this disease [28, 29]. In Najafi’s study, the authors evaluated 3591 participants aged higher than 18 years old in Golestan and reported a prevalence of 4.6% [28], and Barahimi and colleagues found percentage of 4.7 in total of 1400 participants aged over 30 years old in Shahreza, a region in central of Iran [29]. However, a more recent study performed by Sepanlou and colleagues in 2017 showed the CKD stage III+ to be as high as 23.7% among 11,000 participants enrolled in the second phase of the Golestan cohort study who aged between 40 and 75 years [11]. In a recent systematic review that included more than 70,000 individuals across our country, the overall prevalence of CKD of all ages was 11.6%, while this value reached 24.43% for people aged higher than 40 years [30]. We have to emphasize that we cannot draw any conclusion regarding these variations in the CKD report nor could completely compare them to our study due to the variable settings of studies [26, 28, 31, 32]. However, we may emphasize that the relatively lower rate of CKD stage III+ prevalence seen in our study compared to other studies might be explained, by the younger age of included subjects in addition to exclusion of the elderly (age > 65 years). Accordingly, only 10% of our CKD stage III+ subjects aged less than 40 years while we observed a steady rise in CKD prevalence with an increase in age. Other than being an important risk factor, the strong association of age and CKD stage III+ prevalence in this study has a crucial implication in health policy and prevention, since within a few decades we might expect a huge rise in the CKD prevalence as the proportion of elderly people increases in this province.

Estimation of GFR is a crucial part of assessing kidney function for reporting the CKD prevalence in population studies. We need to stress that we cannot completely compare our study with some other studies, because of the different method of applying two equations instead of one (which presents a novel structure) and also because we only focused on CKD stage III+. The two most employed equations for GFR estimation are MDRD and CKD-EPI. The original 1999 MDRD study equation derived from Caucasians and African Americans with CKD [12], However, the equation was modified over time [33]. The CKD-EPI was formed in 2009 by the National Institute of Diabetes and Digestive and Kidney Diseases by gathering pooled datasets from diverse studies [13]. Many regional published studies have evaluated the CKD prevalence based on the MDRD equation. However, in this study, we employed both equations separately and also in combination to define the CKD stage III+ prevalence and found a lower rate of CKD stage III+ prevalence with the CKD-EPI equation. It is suggested that the MDRD formula is more accurate for estimating GFR when its value is less than 60 mL/min/1.73 m^2^, because the relation between serum creatinine and eGFR varies in healthy and CKD population and that in healthy populations MDRD study equation probably systematically underestimates GFR, leading to higher rates of CKD mostly in females and youngsters [15, 16, 34, 35]. This is in accordance with our observation that CKD stage III+ was more prevalent in female subjects when the MDRD equation was applied (7.27% versus 6.82%). This trend was vice versa when the CKD-EPI equation was used (5.07% versus 6.27%).

In this study, we were able to investigate the association of many risk factors with CKD stage III+. Our study confirmed the relation between traditional risk factors including age, hypertension, low levels of HDL in addition to increasing in the waist to hip ratio with CKD stage III+ probability [10, 11, 28, 31, 36, 37]. This study similar to other studies found DM as a determinant for CKD stage III+ in crude analysis [10, 11, 27]. However, the role of DM was faded in the adjusted model. We cannot give a conclusion on the reason behind this finding, but lack of the data on the onset of CKD and diabetes might be considered.

The predictivity of anthropometric indices for CKD probability in previous studies is controversial. Noori and colleagues reported that waist circumferences are a better predictive index in CKD among other anthropometric indices [38], while Sepanlou and colleagues found the waist to height ratio as the most proper anthropometric index for CKD prediction [11]. Similarly, waist to hip ratio was the best predictor of CKD stage III+ among anthropometric indices in our study showing an OR of 1.31 in multivariable analysis.

Previous studies reported that women are at greater risk of CKD [11, 27, 32, 39, 40], however, in this study we could not confirm such an association between gender and CKD stage III+. It is possible that the relationship between being female and the risk of CKD stage III+, observed in other studies, be partly related to residual confounding from high BMI, lower physical activity, and subclinical diabetes [28], or using MDRD equation that reports higher CKD stage III+ prevalence in females. A robust response needs further studies and evaluations.

Around half of the included subjects in this study had Arab ethnicity. The prevalence of CKD in Arabian countries in the middle east was reported between 3.5 - 6.6% in 2017 [2]. A rate that is relatively lower compared to other countries. Accordingly, in this study, we found that Arab ethnicity acts as a protective factor against CKD stage III+. Assessing the underlying cause of this relation and the exact role of ethnicity on CKD was beyond the scope of this study, however, this novel finding can open the path for further ethnical studies in the region. Noteworthy the diet with low to moderate carbohydrate are more popular among Arabs, while the rice considers as the main dish of Fars. The bread and red meat comprise the main meal of Bakhtiaries and Lurs. Whether these differences in lifestyle contributed to the lower rate of CKD among Arab ethnicity needs to be answered in future studies.

We also found an association between a higher wealth score and CKD stage III+ probability. In general, individuals in lower socioeconomic status groups may suffer more frequently from CKD [41]. However, in some settings this association may be weakened or reversed [42–44]. It is plausible that individuals with higher income are more likely to have a western lifestyle, such as type of food consumption, alcohol use, smoking, and sedentary lifestyle, and consequently, in those setting people with higher wealth scores have higher CKD risk.

In this study, we found that 27% of subjects with CKD stage III+ had DM while when we looked at all the participants, the prevalence of diabetes was 15.4%. In third national surveillance of risk factors of non-communicable diseases, the prevalence of DM in our country estimated to be 8.7% [45]. In 2014, the highest prevalence of DM in Eastern Mediterranean region, reported by WHO, was 13.7% which is close to our finding in this report. On the other hand, the prevalence of DM seems to be lower in Europe (7.3%) and North America (8.3%). While all the regions have experienced a double change in the prevalence of diabetes from 1980, change in the middle east seems to be more prominent. The upward trends of diabetes growth in middle east can be due to urbanization, more consumption of processed food, and lower states of physical activity. Another reason for relatively lower number of subjects with diabetes could be related to our method of diabetes definition that implied only one- time blood sugar measurement which could result in diagnosis of lower number of subjects with diabetes.

The ischemic heart disease in this study was defined based on “subject’s report”. We assume that this could be the reason behind the high prevalence of IHD among these populations. This variable lost its association with CKD in multivariate analysis. Although cannot be conclusive but this finding may further stress that the real number of subjects with IHD might be lower than what we found in this study.

Our survey has both strengths and limitations. The important strength of our study was the large number of included subjects with relatively comprehensive collected data in a population-based study. Another strength is that we applied both equations for identifying the CKD stage III+ group. This provides a comparison of two equations in the same population and may provide more robust results in identifying the CKD determinants. The first limitation of our study is that the population included in the study were from the southwest region of our country and therefore our study cannot be regarded as a nationwide study. Lack of urinary albumin and protein excretion data was another limitation. It is plausible that true prevalence of CKD would be higher than what we obtained in this study. Because we did not have the urine data so we could not identify the CKD stages I, II and subjects with urine sediment impairment without creatinine change. The third limitation is that we identified participants to have CKD stage III+ with only one serum creatinine measurement so we cannot rule out the possibility that not all the identified subjects with CKD stage III+ had a permanent impaired renal function. The major causes for not having two repeated measurements of serum creatinine in this study were: budget limitation: the study was designed and financially supported as a cross sectional study; high rate of drop out: based on previous experiments the rate of drop out is high when the subjects were required to have 2 blood drawn over a relatively short period of time (3 months). The fourth limitation is applying CKD-EPI formula for estimating GFR on serum creatinine based on Jaffe method. Since IDMS method is not available in Iran, we were forced to use the only available method of creatinine measurement. The unavailable IDMS method in Iran pushes back nephrologists and researchers in appliance of the up-to-date formula in Iranian GFR estimation. However, this study can be used as a reference for comparison of the IDMS based GFR estimation and validation of the formula on Iranian population in the future. Finally, executive problems to re-recruit a large number of subjects after 3 months from 27 counties. The study is part of a larger study (KCHS), which aimed to grasp an overview on non-communicable diseases. We plan to conduct disease-oriented cohorts in the region after summarizing the whole status of the region, in which CKD will be launched in a more accurate method.

## Conclusion

In summary, CKD has become an important health problem in our country that produces a huge financial and social burden on the national health system. The findings of the present study provided a summary of CKD stage III+ prevalence and its determinants in southwest part of our country and confirmed the results of the previous studies on risk factors and determinants of low eGFR including, hypertension, age, and lipid abnormality. Consequently, aging of the population as well as the increasing trend of other risk factors of CKD among our population worsen the problem. Identifying the CKD risk factors, early diagnosis, and providing the appropriate care is crucial in decreasing the mortality and morbidity and can slow down the progression toward ESRD.

## Supplementary Information

**Additional file 1.**

## Data Availability

The datasets used and analyzed during the current study are available from the corresponding author on reasonable request.

## Abbreviations

CKD: chronic kidney disease
KCHS: the Khuzestan Comprehensive Health Study
MDRD: Modification of Diet in Renal Disease
CKD-EPI: Chronic Kidney Disease Epidemiology Collaboration
eGFR: estimated glomerular filtration rate
ESRD: end-stage renal disease
NIMAD: National Institute for Medical Research Development
IBTO: Iranian Blood Transfusion Organization
DDRI: Digestive Diseases Research Institute
IPAQ: International Physical Activity Questionnaires
FBS: fasting blood sugar
Cr: creatinine
TC: total cholesterol
TG: triglyceride
HDL: and high-density lipoprotein
HTN: Hypertension
SBP: systolic blood pressure
DBP: diastolic blood pressure
DM: Diabetes mellitus
MET: metabolic equivalent task
IDMS: isotope dilution mass spectroscopy
Hyperchol: Hypercholesteremia
IHD: Ischemic Heart Disease
MS: Metabolic Syndrome
BMI: Body Mass Index
WC: Waist circumference
WHtR: Waist to height ratio
WHR: Waist to hip ratio
