# Supplementary material for: Prevalence of impaired renal function and determinants in the southwest of Iran
Source: BMC Nephrol. 2021 Aug 10;22:276. doi: 10.1186/s12882-021-02484-x (PMC8353841; doi:10.1186/s12882-021-02484-x)
Supplement: Supplementary file 1 — Additional file 1. [file 12882_2021_2484_MOESM1_ESM.pdf]

IBTO DUMS BehUMS AbadanUMS AJUMS DDRI

Questionnaire for assessing physical health status in Khuzestan province

Date of interview:	……. / ……. / 20 …
Place of interview:	Province: ....<br>City: ....<br>Township: ....<br>Village: ....<br>Health center: ....
Questioner:	Name: ....<br>Family name: ....<br>Signature: ....

ID- The unique KCHS ID for each participant

Province code	Township code	Area of residence code	Family code	Person code in family
☐☐	☐☐	☐	☐☐☐☐	☐☐

GI- General information

G1. Name: ……………………………………………..	G8. Occupation: ..……………………………………..
G2. Father’s name: ……………………………………	G9. Telephone and cellphone number: ………………..
G3. National code: ……………………………………	G10. Address: …………………………………………
G4. Postal code: ………..……………………………	G11. Gender: Female ☐ ……..….. Male ☐ …………
G5. E-mail: ……………………………………………	G12. Birth date: …… / ……. / …….
G6. Education years: (regardless of fails): ……………	G13. Marital status: Single ☐ …….. Married ☐ ……..<br>Divorced ☐ ….. Widowed ☐ …..
G7. Last educational establishment: ………………......	G14. Insurance type: Uninsured ☐ …. Insured ☐ ….

GFI- General family information (Not used in this study)

PH/E- Physical examinations and anthropometric measurements

P1.	P2.	P3.	P4.	P5.	P6.	P7.
Height (Cm)	Weight (Kg)	Waist circ. (Cm)	Hip circ. (Cm)	Wrist circ. (Cm)	SBP	DBP
..…. / ……	..…. / ……	..…. / ……	..…. / ……	..…. / ……	……	……

S- Sleep

S1	What time do you usually go to sleep?	….. : …...
S2	What is the average number of minutes it takes you to fall asleep at night?	….. min
S3	What time do you usually get up?	….. : …...
S4	What time do you prefer to get up?	….. : …...
S5	Do you nap during the day? (3 times a week or more)	Yes ☐ No ☐<br>If yes: …. min
S6	Do you do night shift work? (now or in the past year, for at least 6 hours from 9pm - 6am.)	Yes ☐ No ☐<br>If yes: …. times
S7	While sleeping, do you have restless leg movements so that your partner notices?	Yes ☐ No ☐<br>I don't know ☐
S8	When doing nothing, are likely are you to doze off or fall asleep?	Yes ☐ No ☐
S9	Do you often use hypnotics when you sleep? (2 times a week or more)	Yes ☐ No ☐

IPAQ- International physical activity questionnaire

**Part 1. Job-related physical activity:** The first section is about your work. This includes paid jobs, farming, volunteer work, course work, and any other unpaid work that you did outside your home. Do not include unpaid work you might do around your home, like housework, yard work, general maintenance, and caring for your family. These are asked in Part 3.		
PA1	Do you currently have a job or do any unpaid work outside your home?<br>Yes ☐<br>No ☐ → Skip to part 2	
PA2	During the last 7 days, on how many days did you do vigorous physical activities like heavy lifting, digging, heavy construction, or climbing up stairs as part of your work? Think about only those physical activities that you did for at least 10 minutes at a time.<br>….. days per week.<br>No vigorous job-related physical activity. ☐ → Skip to question 4	

PA3	How much time did you usually spend on one of those days doing vigorous physical activities as part of your work?<br>….. hours per day<br>….. minutes per day	
PA4	Again, think about only those physical activities that you did for at least 10 minutes at a time. During the last 7 days, on how many days did you do moderate physical activities like carrying light loads as part of your work? Please do not include walking.<br>….. days per week<br>No moderate job-related physical activity. ☐ → Skip to question 6	
PA5	How much time did you usually spend on one of those days doing moderate physical activities as part of your work?<br>….. hours per day<br>….. minutes per day	
PA6	During the last 7 days, on how many days did you walk for at least 10 minutes at a time as part of your work? Please do not count any walking you did to travel to or from work.<br>….. days per week<br>No job-related walking. ☐ → Skip to part 2	
PA7	How much time did you usually spend on one of those days walking as part of your work?<br>….. hours per day<br>…… minutes per day	
**Part 2. Transportation physical activity:** These questions are about how you traveled from place to place, including to places like work, stores, movies, and so on.		
PA8	During the last 7 days, on how many days did you travel in a motor vehicle like a train, bus, car, or tram?<br>….. days per week<br>No traveling in a motor vehicle. ☐ → Skip to question 10	
PA9	How much time did you usually spend on one of those days traveling in a train, bus, car, tram, or other kind of motor vehicle?<br>….. hours per day<br>….. minutes per day	
Now think only about the bicycling and walking you might have done to travel to and from work, to do errands, or to go from place to place.		
PA10	During the last 7 days, on how many days did you bicycle for at least 10 minutes at a time to go from place to place?<br>….. days per week<br>No bicycling from place to place. ☐ → Skip to question 12	
PA11	How much time did you usually spend on one of those days to bicycle from place to place?<br>….. hours per day<br>….. minutes per day	
PA12	During the last 7 days, on how many days did you walk for at least 10 minutes at a time to go from place to place?<br>….. days per week<br>No walking from place to place. ☐ → Skip to part 3	

PA13	How much time did you usually spend on one of those days walking from place to place?<br>….. hours per day<br>….. minutes per day	
**Part 3. Housework, house maintenance, and caring for family:** This section is about some of the physical activities you might have done in the last 7 days in and around your home, like housework, gardening, yard work, general maintenance work, and caring for your family.		
PA14	Think about only those physical activities that you did for at least 10 minutes at a time. During the last 7 days, on how many days did you do vigorous physical activities like heavy lifting, chopping wood, shoveling snow, or digging in the garden or yard?<br>….. days per week<br>No vigorous activity in garden or yard. ☐ → Skip to question 16	
PA15	How much time did you usually spend on one of those days doing vigorous physical activities in the garden or yard?<br>….. hours per day<br>….. minutes per day	
PA16	Again, think about only those physical activities that you did for at least 10 minutes at a time. During the last 7 days, on how many days did you do moderate activities like carrying light loads, sweeping, washing windows, and raking in the garden or yard?<br>….. days per week<br>No moderate activity in garden or yard. ☐ → Skip to question 18	
PA17	How much time did you usually spend on one of those days doing moderate physical activities in the garden or yard?<br>….. hours per day<br>….. minutes per day	
PA18	Once again, think about only those physical activities that you did for at least 10 minutes at a time. During the last 7 days, on how many days did you do moderate activities like carrying light loads, washing windows, scrubbing floors and sweeping inside your home?<br>….. days per week<br>No moderate activity inside home. ☐ → Skip to part 4	
PA19	How much time did you usually spend on one of those days doing moderate physical activities inside your home?<br>….. hours per day<br>….. minutes per day	
**Part 4. Recreation, sport, and leisure-time physical activity:** This section is about all the physical activities that you did in the last 7 days solely for recreation, sport, exercise or leisure. Please do not include any activities you have already mentioned.		
PA20	Not counting any walking, you have already mentioned, during the last 7 days, on how many days did you walk for at least 10 minutes at a time in your leisure time?<br>….. days per week<br>No walking in leisure time. ☐ → Skip to question 22	
PA21	How much time did you usually spend on one of those days walking in your leisure time?<br>….. hours per day<br>….. minutes per day	

PA22	Think about only those physical activities that you did for at least 10 minutes at a time.<br>During the last 7 days, on how many days did you do vigorous physical activities like aerobics, running, fast bicycling, or fast swimming in your leisure time?<br>….. days per week<br>No vigorous activity in leisure time. ☐ → Skip to question 24	
PA23	How much time did you usually spend on one of those days doing vigorous physical activities in your leisure time?<br>….. hours per day<br>….. minutes per day	
PA24	Again, think about only those physical activities that you did for at least 10 minutes at a time. During the last 7 days, on how many days did you do moderate physical activities like bicycling at a regular pace, swimming at a regular pace, and doubles tennis in your leisure time?<br>….. days per week<br>No moderate activity in leisure time. ☐ → Skip to part 5	
PA25	How much time did you usually spend on one of those days doing moderate physical activities in your leisure time?<br>….. hours per day<br>….. minutes per day	
**Part 5. Time spent sitting:**<br>The last questions are about the time you spend sitting while at work, at home, while doing course work and during leisure time. This may include time spent sitting at a desk, visiting friends, reading or sitting or lying down to watch television. Do not include any time spent sitting in a motor vehicle that you have already told me about.		
PA26	During the last 7 days, how much time did you usually spend sitting on a weekday?<br>….. hours per day<br>….. minutes per day	
PA27	During the last 7 days, how much time did you usually spend sitting on a weekend day?<br>….. hours per day<br>….. minutes per day	

RH- Female and male reproductive history (Not used in this study)

PMH- Past medical history of chronic diseases (Only the ones that were used in this study)

PMH1	Diabetes	Yes ☐ No ☐	Onset age: …..	Less than a year. ☐
PMH2	HTN	Yes ☐ No ☐	Onset age: …..	Less than a year. ☐
PMH3	HLP	Yes ☐ No ☐	Onset age: …..	Less than a year. ☐
PMH4	Malignancy	Yes ☐ No ☐<br>Type: …………...	Onset age: …..	Less than a year. ☐
PMH5	Chronic kidney disease<br>Dialysis<br>Kidney transplantation	Yes ☐ No ☐<br>Yes ☐ No ☐<br>Yes ☐ No ☐	Onset age: …..<br>Onset age: …..<br>Onset age: …..	Less than a year. ☐<br>Less than a year. ☐<br>Less than a year. ☐

HH- Habitual history (Only the ones that were used in this study)

HH1	Do you currently smoke?	Yes No	For how long? ……………………
HH2	If not, have you ever smoked in the past?	Yes No	For how long? ……………………
HH3	Do you currently smoke hookah?	Yes No	For how long? ……………………
HH4	If not, have you ever smoked hookah in the past?	Yes No	For how long? ……………………
HH5	Do you currently use opium?	Yes No	For how long? ……………………
HH6	If not, have you ever used opium in the past?	Yes No	For how long? ……………………
HH7	Do you currently drink alcohol?	Yes No	For how long? ……………………
HH8	If not, have you ever drunk alcohol in the past?	Yes No	For how long? ……………………

DH- Drug history

Drug name	For how long

FH- Family history (Not used in this study)

DT- Factors associated with disease transmission (Not used in this study)

SES- Socioeconomic status

SES1	What is the tenure status of the home used by the household?<br>1. Ownership 2. Rent 3. Organizational (governmental or private) 4. Kinship (parents or relatives with no renting contract) 5. Others	
SES2	What is the area of the residential unit? (Regardless of garden, porch, workshop, yard, and where the animals are kept)?<br>….. m^2	
SES3	Number of rooms in current home? (Regardless of living room and kitchen?<br>….. rooms	
SES4	How many family members live together in your current home?<br>….. members	

SES5	Which of the following items do your home own?<br><br>1. Separate freezer: Yes □ No □<br>2. Computer/laptop: Yes □ No □<br>3. Washing machine: Yes □ No □<br>4. Internet access: Yes □ No □<br>5. Dishwashing machine: Yes □ No □<br>6. Motorcycle: Yes □ No □<br>7. Indoor bathroom/shower: Yes □ No □<br>8. Color TV: Yes □ No □, If yes: Ordinary □<br>Plasma □<br>9. Vacuum cleaner: Yes □ No □<br>10. Car: Yes □ No, If yes: <20 million Toman □<br>20-50 million Toman □<br>50-100 million Toman □<br>>100 million Toman □	
SES6	Which of the following items do the home occupants own?<br><br>1. Cellphone: Yes □ No □<br>2. Laptop: Yes □ No □<br>3. Computer: Yes □ No □<br>4. Internet Access: Yes □ No □<br>5. Car: Yes □ No, If yes: <20 million Toman □<br>20-50 million Toman □<br>50-100 million Toman □<br>>100 million Toman □	
SES7	The number of extracurricular and non-professional books that you have read in the previous year (excluding scriptures and prayers)?<br>….. books	
SES8	The number of trips to foreign countries in a lifetime?<br>….. pilgrimage<br>….. non-pilgrimage<br>Never □	
SES9	The number of internal trips in the last ten years (pilgrimage or tourism that is at least 100 km away from the residence)?<br>….. trips<br>Never □	

Psychology survey- LEC, PCL, War and DASS (Not used in this study)
